# Forensic characteristics and population genetics of Chinese Kazakh ethnic minority with an efficient STR panel

**DOI:** 10.7717/peerj.6802

**Published:** 2019-04-25

**Authors:** Chong Chen, Yuxin Guo, Xiaoye Jin, Wei Cui, Yuanyuan Wei, Yating Fang, Qiong Lan, Tingting Kong, Tong Xie, Bofeng Zhu

**Affiliations:** 1 Key Laboratory of Shaanxi Province for Craniofacial Precision Medicine Research, College of Stomatology, Xi’an Jiaotong University, Xi’an, Shaanxi, China; 2 Clinical Research Center of Shaanxi Province for Dental and Maxillofacial Diseases, College of Stomatology, Xi’an Jiaotong University, Xi’an, Shaanxi, China; 3 College of Medicine and Forensics, Xi’an Jiaotong University Health Science Center, Xi’an, China; 4 Department of Forensic Genetics, School of Forensic Medicine, Southern Medical University, Guangzhou, Guangdong, China

**Keywords:** Non-CODIS STRs, Chinese Kazakh, Forensic population genetics, Geneticpolymorphism

## Abstract

On the purpose of enhancing the forensic efficiency of CODIS STR loci, new STR loci have been gradually discovered and developed into some commercial multiplex systems. Recently, 22 STR loci including 18 non-CODIS STR loci and four CODIS STR loci were investigated in 501 unrelated healthy individuals of Kazakh ethnic group. Seven to 20 alleles at the different loci were identified and altogether 276 alleles for 22 selected loci were detected with the corresponding allelic frequencies ranging from 0.0010 to 0.3623. No significant deviation was observed from the Hardy–Weinberg equilibrium test for any of the 22 STRs. The value of cumulative power of discrimination in Kazakh group was 1-1.00E^−28^. Analyses of population differentiations and genetic distances between Kazakh and other Chinese groups presented that the Kazakh group with the Uygur group. These 22 STR loci evenly distributed on 22 different autosomal chromosomes were characterized by high genetic diversities and therefore could be utilized in the forensic cases to further increase the discrimination performance.

## Introduction

Since the Federal Bureau of Investigation laboratory selected 13 autosomal STR loci as core loci of Combined DNA Index System (CODIS) in 1997, STR loci from CODIS have been applied into the forensic applications and commonly contained in many commercial kits (Gonçalves et al., 2002; Lan et al., 2018; Wang et al., 2012; Xiao et al., 2018). It is worth noting that seven new markers were added up to these 13 core STR loci, creating an expanded panel with 20 CODIS core loci in 2015 (Hares, 2015). However, in forensic applications, it is indispensable to combine the non-CODIS STRs with the previously commercial CODIS STR kits in order to increase the discrimination power (Inokuchi et al., 2018; Kuzniar, Jastrzebska & Ploski, 2006; Tsai et al., 2013) in some cases, like missing person investigations, complicated parentage testing cases and those with mutation events. In this study, 22 selected loci (Li et al., 2017) were introduced containing 18 non-CODIS STR loci and four CODIS STR loci in which three (D1S1656, D2S1338 and D12S391) were recognized as CODIS core loci in 2015 (Hares, 2015) and one (D16S539) belonged to 13 CODIS core loci.

The Kazakh group is one of the minority nationalities of China, with a population exceeding 1.46 million people (data derived from the sixth National Population Census of China, 2010) dwelling in the Xinjiang Uygur Autonomous Region, Qinghai and Gansu provinces. There have been some research focused on the Kazakh group from Xinjiang Uygur Autonomous Region (Liu et al., 2017; Mei et al., 2016; Yuan et al., 2014; Zhang et al., 2016), but very few studies were available for non-CODIS STRs in the Kazakh group. To enrich the database of population genetics and probe into the genetic background of Kazakh group, we calculated the allelic frequency distributions as well as forensic parameters of these 22 autosomal STRs for the first time in Kazakh group, and conducted comparative analysis with other five Chinese reference groups.

## Materials and Methods

### The collection of population samples

Whole blood samples were withdrawn from 501 unrelated healthy individuals of Chinese Xinjiang Kazakh group. During the process of sample collection, we ensured the genetic relationships by inquiring and no blood relationship was existed between any two individuals at least in three generations. Moreover, all individuals should meet the requirements of being the aboriginal inhabitants. The research was officially authorized by the ethics committee of Xi’an Jiaotong University Health Science Center, China (Ethical Application No: 2018-518) before the study, and was performed as per the human and ethical research principles of Xi’an Jiaotong University Health Science Center. All the participators signed informed consent statements prior to the specimen collections.

### Multiplex amplification and STR genotyping

DNA was directly amplified without extraction based on a multiplex PCR system that simultaneously amplified 22 STR loci from Microreader™ 23sp ID kit. PCR amplification was implemented on the GeneAmp^®^ PCR 9700 thermocycler (Applied Biosystems, Foster City, CA, USA) with 25 µL reaction volume, and amplified products were subsequently separated and analyzed on the ABI PRISM^®^ 3130XL Genetic Analyzer (Applied Biosystems, Foster City, CA, USA) with reference to internal lane standard Org500 (including different length fragments: 50, 75, 100, 139, 150, 160, 200, 300, 340, 350, 400, 450, 490 and 500 bp). The specific reaction system and conditions for electrophoresis were referred to the previously published research (Li et al., 2017). The analyzation of electrophoresis results were revealed by GeneMapper^®^ ID-X 1.3 software (Applied Biosystems, Foster City, CA, USA).

### Population genetic and forensic statistical analyses

For the studied Kazakh group, the distributions of allelic frequencies and forensic statistical parameters containing the observed heterozygosity (Ho), power of exclusion (PE), power of discrimination (PD), polymorphism information content (PIC) and Hardy–Weinberg equilibrium (HWE) of 22 STR loci were calculated by the modified Powerstats software v.1.2 (Tereba, 1999). Expected heterozygosity (He) was calculated as described by Nei (1978). The estimates of linkage disequilibrium (LD) (Slatkin, 2008) of 22 STR loci were performed by Genepop v.4.0.10 (http://genepop.curtin.edu.au/). Correlation coefficients (*r*^2^) for all allele combinations were ascertained by SHEsis online tool (Yongyong & Lin, 2005). The locus-by-locus fixation index (*Fst* values) and corresponding probability (*p*) values of population genetic differentiations were estimated by Arlequin software v.3.5 (Excoffier & Lischer, 2010) using the analysis of molecular variance (AMOVA) method. The DISPAN program (Ota, 1993) was implemented to calculate Nei’s genetic distance (DA) values (Nei, 1972) utilizing the raw STR genotyping data of Kazakh group. The population pairwise genetic differentiation *Fst* values and *p* values were carried out by Arlequin software v.3.5 (Excoffier & Lischer, 2010) based on raw STR genotyping data. The heat maps of population pairwise *D*_*A*_ and *Fst* values were conducted using *R* statistical software v3.0.2 (Dean & Nielsen, 2007). The description of population genetic structure was presented by the STRUCTURE software v.2.3.4 (Pritchard, Stephens & Donnelly, 2000) and the optimum *K* value, the number of hypothetical ancestral populations, was estimated by Structure Harvester v.0.6.94 (Pritchard, Stephens & Donnelly, 2000).

## Results

### Hardy–Weinberg equilibrium and linkage disequilibrium analyses

HWE tests were performed and the results showed no significant deviations from HWE at these 22 STR loci in Kazakh group after Bonferroni correction (Curtin & Schulz, 1998) (*p* = 0.05∕22 = 0.00227273). In Fig. S1, the locations of STR loci were indicated on each autosomal chromosome. Furthermore, as shown in Table S1, significant *p*-values for LD analyses were detected in D10S1435-D15S659 and D13S325-D17S1290 after applying Bonferroni correction (Curtin & Schulz, 1998) (*p* = 0.05∕231 = 0.00021645). The *r*^2^ values of 22 STR loci were displayed in the Fig. S2. The result transpired that the *r*^2^ values of STR pairs were all less than 0.004.

**Table 1:** The allelic frequencies for 22 autosomal STR loci in Xinjiang Kazakh population (*n* = 501).

Alleles	D1S1656	D2S1338	D3S3045	D4S2366	D5S2500	D6S477	D7S3048	D8S1132	D9S925	D10S1435	D11S2368	D12S391	D13S325	D14S608	D15S659	D16S539	D17S1290	D18S535	D19S253	D20S470	D21S1270	D22-GATA198B05
**4**														0.0070								
**5**														0.0010								
**6**														0.0230						0.0329		
**7**										0.0010				0.2116		0.0010			0.1936			
**8**	0.0010		0.0050							0.0080				0.0240		0.0190		0.0020	0.0250	0.0050		
**9**			0.2974	0.2804	0.0030					0.0020				0.0858	0.0090	0.2166	0.0030	0.1996	0.0130	0.0060	0.0030	
**9.2**						0.0080																
**10**	0.0030		0.0369	0.0649	0.0778	0.0020			0.0040	0.0150				0.2265	0.0549	0.1397	0.0190	0.0200	0.0230	0.1377	0.2515	
**10.2**						0.0220																
**10.3**										0.0020										0.0010		
**11**	0.0689		0.0519	0.2295	0.2784	0.0140			0.0349	0.1257				0.2315	0.1367	0.2196	0.0269	0.0210	0.1068	0.0209	0.0579	
**11.2**						0.0050																
**11.3**										0.0010												
**12**	0.0698		0.1487	0.1637	0.1976	0.0489			0.0210	0.3623				0.1327	0.1826	0.2485	0.0060	0.1317	0.3243	0.0469	0.0499	0.0020
**12.1**								0.0010														
**12.2**						0.0070				0.0080												
**12.3**																				0.0070	0.0539	
**13**	0.0629		0.2395	0.1317	0.0649	0.1776			0.0050	0.2325			0.0010	0.0559	0.1228	0.1287	0.0060	0.2794	0.2265	0.1507	0.1237	
**13.2**						0.0020																
**13.3**																				0.0090	0.0529	
**14**	0.0858		0.1647	0.1118	0.0629	0.2116			0.1088	0.2156				0.0010	0.0339	0.0269	0.0140	0.2675	0.0788	0.1617	0.2645	0.0050
**14.2**						0.0010																
**14.3**	0.0010																			0.0030	0.0309	
**15**	0.2665		0.0529	0.0180	0.2305	0.2754			0.2335	0.0209	0.0010	0.0100	0.0040		0.1996		0.2515	0.0698	0.0070	0.1756	0.0948	0.0240
**15.3**	0.0190								0.0020											0.0030	0.0030	
**16**	0.2076	0.0100	0.0030		0.0659	0.1826	0.0020	0.0030	0.3343	0.0050	0.0349	0.0160	0.0060		0.1427		0.3223	0.0090	0.0020	0.1527	0.0140	0.0778
**16.1**																				0.0010		
**16.3**	0.0249								0.0060	0.0010												
**17**	0.0768	0.0938			0.0180	0.0289	0.0140	0.0479	0.1896		0.1417	0.1157	0.0130		0.1008		0.1567			0.0589		0.0978
**17.3**	0.0559											0.0130										
**18**	0.0060	0.1068			0.0010	0.0070	0.0998	0.1717	0.0589		0.0908	0.2086	0.0749		0.0160		0.1337			0.0170		0.0938
**18.1**												0.0010										
**18.2**												0.0020										
**18.3**	0.0369											0.0100										
**19**		0.1946				0.0050	0.0738	0.1926	0.0010		0.1787	0.1916	0.2864		0.0010		0.0429			0.0100		0.1068
**19.3**	0.0130											0.0030										
**20**		0.1237				0.0020	0.1267	0.1307	0.0010		0.1467	0.1647	0.2395				0.0110					0.1298
**20.3**	0.0010																					
**21**		0.0239					0.1218	0.1208			0.2525	0.0778	0.1896				0.0050					0.2355
**21.3**												0.0010										
**22**		0.0479					0.0828	0.1647			0.0998	0.0968	0.1357				0.0020					0.1896
**23**		0.1707					0.1637	0.1257			0.0469	0.0409	0.0249									0.0359
**24**		0.1148					0.1557	0.0359			0.0050	0.0279	0.0190									0.0020
**25**		0.0898					0.1098	0.0040				0.0150	0.0030									
**26**		0.0150					0.0399	0.0010			0.0020	0.0030	0.0010									
**27**		0.0040					0.0100	0.0010				0.0010	0.0020									
**28**		0.0050										0.0010										

### Allelic frequencies and forensic parameters of Kazakh group

Raw genotyping data were presented in the Table S2. Allelic frequencies of the 22 STRs in the Kazakh group were enumerated in Table 1. Totally 276 alleles for 22 loci were found with the corresponding allelic frequencies varied from 0.001 to 0.3623. The least number of alleles (seven) was observed at D4S2366 locus, while the maximum (20) was at D12S391 locus. As presented in Fig. S3, the values of PE and Ho were in the range of 0.4903 to 0.7877, and 0.7385 to 0.8962, respectively, with the minimum values detected at D17S1290 locus and maximum at D20S470 locus. Additionally, the minimum values of PD, He and PIC emerged at D10S1435 locus with 0.8993, 0.7523 and 0.7124, separately. Conversely, the maximum values were all observed at D7S3048 locus with 0.9732, 0.8827 and 0.8700, respectively. The cumulative power of discrimination value for all 22 STR loci in the Kazakh group was 1-1.00E^−28^.

### Interpopulation differentiation analyses between Kazakh and reference groups

In order to explore the population genetic differentiations between Kazakh group and the five previously reported reference groups, we calculated the locus-by-locus *Fst* values with *p* values of the 22 loci. As shown in Table 2, significant differences were observed between Kazakh and Xinjiang Uygur (Song et al., 2017) at nine loci, Xinjiang Hui (Fang et al., 2018) at 14 loci, Guangdong Han (Chen et al., 2016) at nine loci, Northern Han (Xie et al., 2015) (Hebei, Henan, Shaanxi) at 18 loci and Hainan Li (Chen et al., 2016) at 17 loci. However, after Bonferroni correction (*p* = 0.05∕110 = 0.00045455), Xinjiang Kazakh had significant differences with Xinjiang Uygur at one loci, Xinjiang Hui at three loci, Northern Han and Hainan Li at ten loci.

**Table 2:** Locus-by-locus *Fst* and *p* values of 22 overlapping loci for allele frequency distribution comparisons between the Kazakh ethnic group and reference groups.

Loci	Xinjiang Uygur		Xinjiang Hui		Northern Han		Guangdong Han		Hainan Li	
	*Fst*	*p*-value	*Fst*	*p*-value	*Fst*	*p*-value	*Fst*	*p*-value	*Fst*	*p*-value
D1S1656	0.0008	0.2082	0.0018	0.0352	0.0020	0.0068	0.0002	0.8006	0.0069	0.0020
D2S1338	−0.0009	1.0000	0.0025	0.0029	0.0032	**0.0000**	0.0035	0.0811	0.0025	0.0694
D3S3045	0.0021	0.0313	0.0009	0.1975	0.0020	0.0108	0.0001	0.7449	0.0089	**0.0000**
D4S2366	0.0009	0.2151	0.0124	**0.0000**	0.0122	**0.0000**	0.0054	0.0264	0.0294	**0.0000**
D5S2500	0.0018	0.0391	0.0044	0.0010	0.0039	**0.0000**	0.0023	0.2590	0.0056	0.0049
D6S477	0.0045	**0.0000**	0.0028	0.0029	0.0009	0.1193	0.0007	0.5748	0.0055	0.0029
D7S3048	0.0006	0.3656	0.0009	0.2209	0.0011	0.0489	−0.0004	0.9580	0.0233	**0.0000**
D8S1132	0.0005	0.3842	0.0040	**0.0000**	0.0057	**0.0000**	0.0081	0.0049	0.0204	**0.0000**
D9S925	0.0002	0.5904	0.0032	0.0098	0.0039	**0.0000**	0.0038	0.1251	0.0027	0.0899
D10S1435	0.0044	0.0039	0.0046	0.0020	0.0046	**0.0000**	0.0042	0.0929	0.0011	0.3969
D11S2368	0.0013	0.0870	0.0021	0.0078	0.0006	0.2297	0.0051	0.0362	0.0046	0.0020
D12S391	0.0017	0.0323	0.0000	0.8475	0.0006	0.2248	0.0046	0.0557	0.0051	0.0039
D13S325	0.0006	0.3011	0.0001	0.6862	0.0044	**0.0000**	0.0047	0.0577	0.0167	**0.0000**
D14S608	0.0030	0.0049	0.0021	0.0244	0.0022	0.0049	0.0109	0.0010	0.0044	0.0117
D15S659	0.0015	0.0557	0.0018	0.0244	0.0028	**0.0000**	0.0073	0.0020	0.0141	**0.0000**
D16S539	0.0002	0.6246	0.0067	**0.0000**	0.0060	**0.0000**	0.0056	0.0440	0.0042	0.0352
D17S1290	0.0006	0.3519	0.0000	0.7341	0.0009	0.1095	0.0035	0.1486	0.0153	**0.0000**
D18S535	0.0037	0.0029	0.0016	0.0528	0.0022	0.0049	0.0105	0.0010	0.0344	**0.0000**
D19S253	−0.0005	1.0000	0.0008	0.2815	0.0021	0.0088	−0.0008	0.9638	0.0024	0.1202
D20S470	0.0007	0.2590	0.0018	0.0205	0.0018	0.0059	0.0098	0.0010	0.0090	**0.0000**
D21S1270	0.0018	0.0332	0.0016	0.0596	0.0021	0.0029	0.0057	0.0313	0.0019	0.1574
D22-GATA198B05	0.0043	0.0010	0.0038	0.0010	0.0052	**0.0000**	0.0038	0.0704	0.0138	**0.0000**

**Notes.**

The numbers in bold indicated the loci showed significant differences between studied Kazakh group and the reference populations after the Bonferroni correction (the significant level =0.05∕110 = 0.00045455).

**Figure 1:**
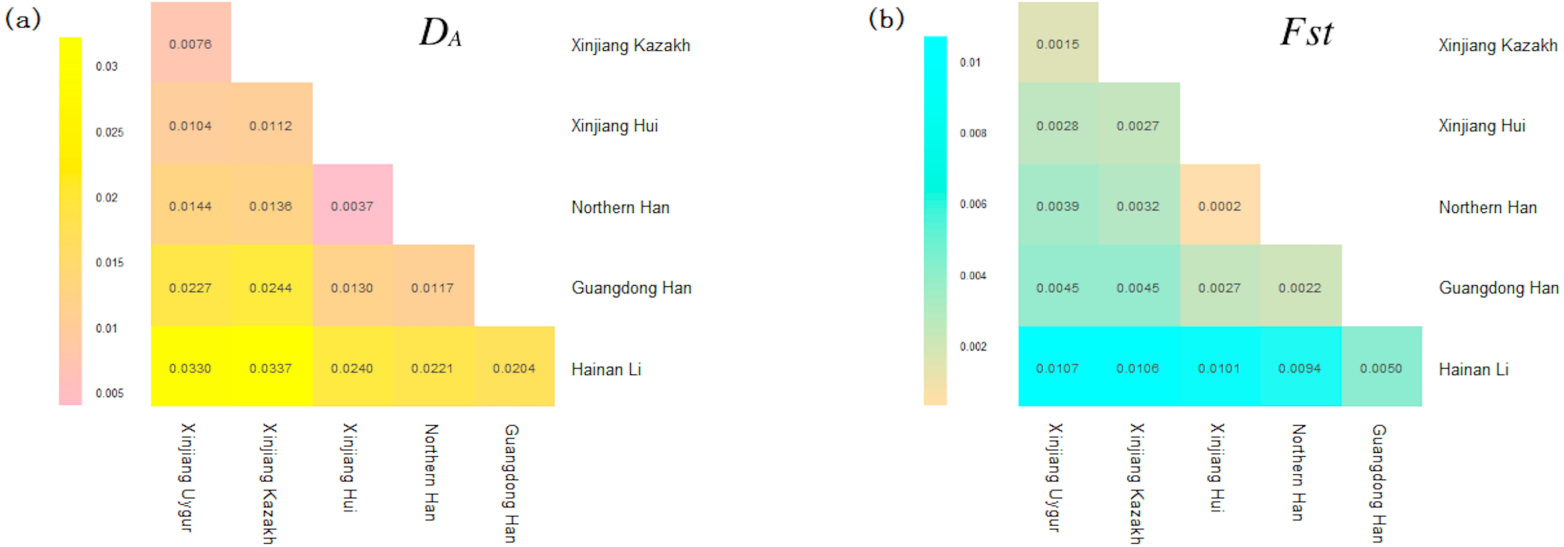
(A) A heat map of pairwise *D*_*A*_ values of Xinjiang Kazakh and five previously published populations based on R software (R Core Team, 2013). (B) A heat map of pairwise *Fst* values of STR loci among Xinjiang Kazakh and five previously published populations conducted by R software.

**Figure 2:**
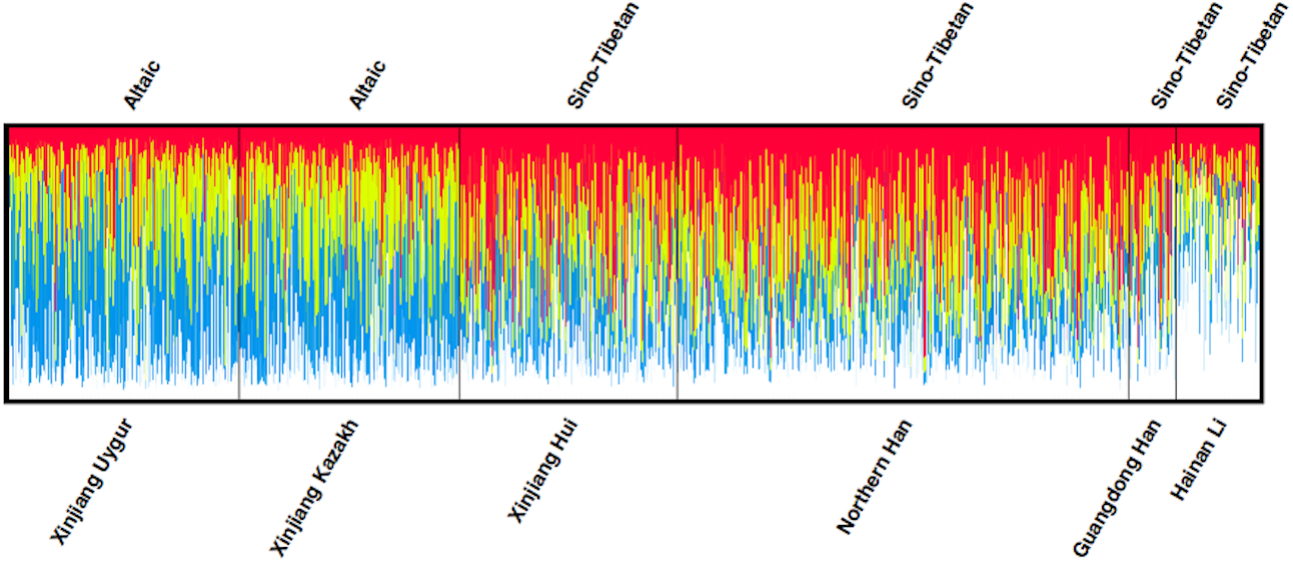
Clustering structure for the full-loci dataset assuming *K* = 4 of the six populations. Structure analysis of six groups, assuming *K* = 4, was presented by the STRUCTURE software v.2.3.4. *K* = 4 was the most suitable number of the estimated ancestral components, which was represented by four colors. The genetic components of each individual were represented by a vertical line divided by colors.

The *D*_*A*_ and *Fst* (*p*) values of pairwise populations were listed in Tables S3 and S4, respectively. Kazakh group had the shortest distance with Uygur group (*D*_*A*_ = 0.0076), followed by Xinjiang Hui (*D*_*A*_ = 0.0112), Northern Han (*D*_*A*_ = 0.0136), Guangdong Han (*D*_*A*_ = 0.0244) and Hainan Li (*D*_*A*_ = 0.0337) groups. The pairwise *Fst* values were also similar to the above *D*_*A*_ results: the studied Kazakh group had the lowest *Fst* value with Xinjiang Uygur group and the highest with Hainan Li group. To visualize the results of population pairwise *D*_*A*_ and *Fst* values more straightforwardly, we performed two heat maps of *D*_*A*_ and *Fst* values between Kazakh and other reference groups as presented in Figs. 1A and 1B. The population structure analyses of the Kazakh and five reference groups were performed using the STRUCTURE software with the result depicted in Fig. 2. *K* = 4 was the most suitable configuration relying on the output posterior probability results (Evanno, Regnaut & Goudet, 2010). Individuals were represented by a vertical line and the colors stood for estimated membership fractions (Rosenberg et al., 2002).

## Discussion

STR loci from CODIS are commonly contained in present commercial kits. However, non-CODIS STRs can be used to enhance discrimination efficiency in some cases (He et al., 2018; Inokuchi et al., 2018). In our study, 22 autosomal STR loci, including 18 non-CODIS loci and four CODIS loci, were utilized to assess their genetic distributions in the Kazakh group living in Xinjiang Uygur Autonomous Region. Significant deviations of LD among these 22 STR loci were detected in D10S1435-D15S659 and D13S325-D17S1290, which were not found in other similar studies based on Microreader™ 23sp ID kit (Fang et al., 2018; Liu et al., 2016). Moreover, the fairly small *r*^2^ values of 22 STR pairs were conductive to determine the low correlations between these pairwise STRs. Hence, these 22 STR loci can be treated as independent markers in Xinjiang Kazakh group. Different ethnic groups in China might have various genetic backgrounds with different allelic frequency distributions, like Han, Xinjiang Kyrgyz and Xibe groups (Guo et al., 2018). Thus, allelic frequency data of these 22 STRs presented here is of great importance for forensic applications in Kazakh ethnic group. In our study, all loci were at a high degree of heterozygosity (He > 0.7, Ho > 0.7) (Akhteruzzaman et al., 2013; Nakamura et al., 1987). PD values of all the loci were greater than 0.9 except for D10S1435 locus (PD = 0.8993), and the PIC values were in the range of 0.7124 to 0.8700. Thus, STR loci in our study could obtain effective information in STR analyses for forensic cases. Besides, this study also enriched the Chinese non-CODIS STRs reference databases.

Various ethnic groups, in China, are deemed to have their special ethnic origins or different genetic backgrounds to some extent (He et al., 2018). In this research, we studied Kazakh group and previously published five Chinese groups with 2,345 individuals to elucidate the population genetic affiliations. The *D*_*A*_ and *Fst* heat maps demonstrated that Kazakh group had closer genetic distances with Xinjiang Uygur group. This result was also supported by the structure analyses. As depicted in Fig. 2, the component of blue color found in Kazakh and Uygur was much higher than that in the other four groups at *K* = 4, the most suitable number of ancestry components.

Our findings were consistent with the previously published studies, such as Y-STR loci described by Shan et al. (2014) and autosomal STR loci depicted by Feng et al. (2018). In Chinese history, although Kazakh and Uygur groups had distinct ethnic origins, both groups all partially encompassed the genetic contributions of the Mongol and Turkic groups (http://www.khazaria.com/genetics/kazakhs.html). Furthermore, Kazakh and Uygur were the main ethnic groups in the ancient Silk Road and they had the similar religious faith, custom and culture (Kong et al., 2017). These historical and cultural backgrounds might explain, at least in part, the minor genetic differentiations between Xinjiang Kazakh and Uygur groups.

## Conclusion

Our results illustrated that these 22 STR loci were highly polymorphic in the Xinjiang Kazakh group and, hence, can be utilized in forensic cases. In addition, close genetic distances and a similar genetic structure demonstrated the intimate genetic relationships between Kazakh and Uygur groups.

##  Supplemental Information

10.7717/peerj.6802/supp-1 Table S1 The pairwise *p*-values of linkage disequilibrium at 22 STRs in Xinjiang Kazakh population

10.7717/peerj.6802/supp-2 Table S2 The raw data of 501 blood samples from Chinese Xinjiang Kazakh group based on 22 STR loci

10.7717/peerj.6802/supp-3 Table S3 The *D*_*A*_ values of pairwise populations based on allelic frequencies of 22 loci among Chinese Xinjiang Kazakh and five reference populations

10.7717/peerj.6802/supp-4 Table S4 The *Fst* values of pairwise populations based on allelic frequencies of 22 loci among Chinese Xinjiang Kazakh and five reference populations

10.7717/peerj.6802/supp-5 Figure S1 The location of 22 STR loci indicated by a short red line on each autosomal chromosome

10.7717/peerj.6802/supp-6 Figure S2 The correlation coefficient (*r*^2^) values of pairwise STRs in Xinjiang Kazakh population

10.7717/peerj.6802/supp-7 Figure S3 The line charts of forensic statistical parameters of 22 STRs in Kazakh population

## Additional Information and Declarations

### Data Availability

The following information was supplied regarding data availability: The raw measurements are available in Table S2.
