# Supplementary figures and images for: Forensic characteristics and population genetics of Chinese Kazakh ethnic minority with an efficient STR panel
Source: PeerJ. 2019 Apr 25;7:e6802. doi: 10.7717/peerj.6802 (PMC6487181; doi:10.7717/peerj.6802)

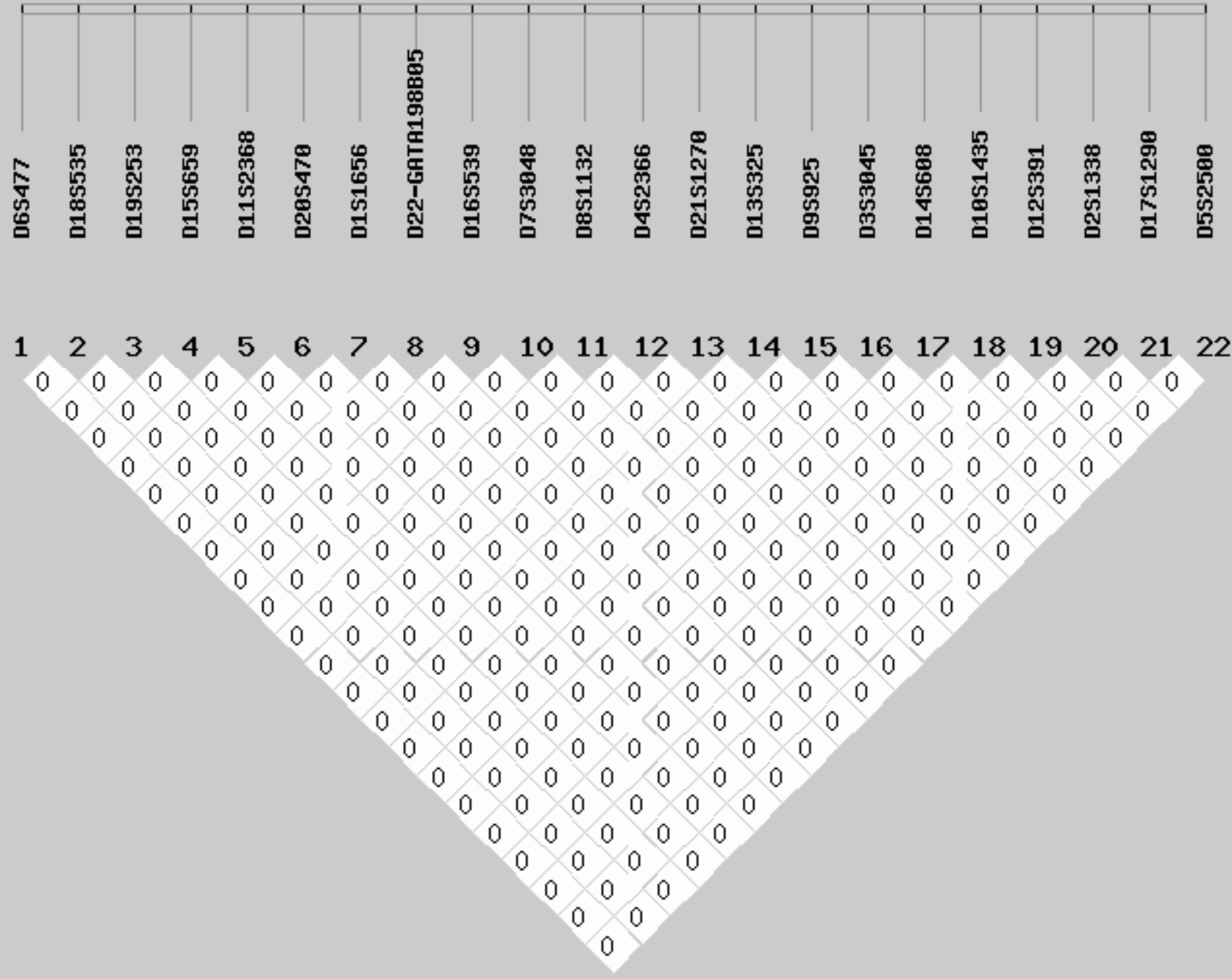

Supplement: Figure S2 [file peerj-07-6802-s006.pdf]

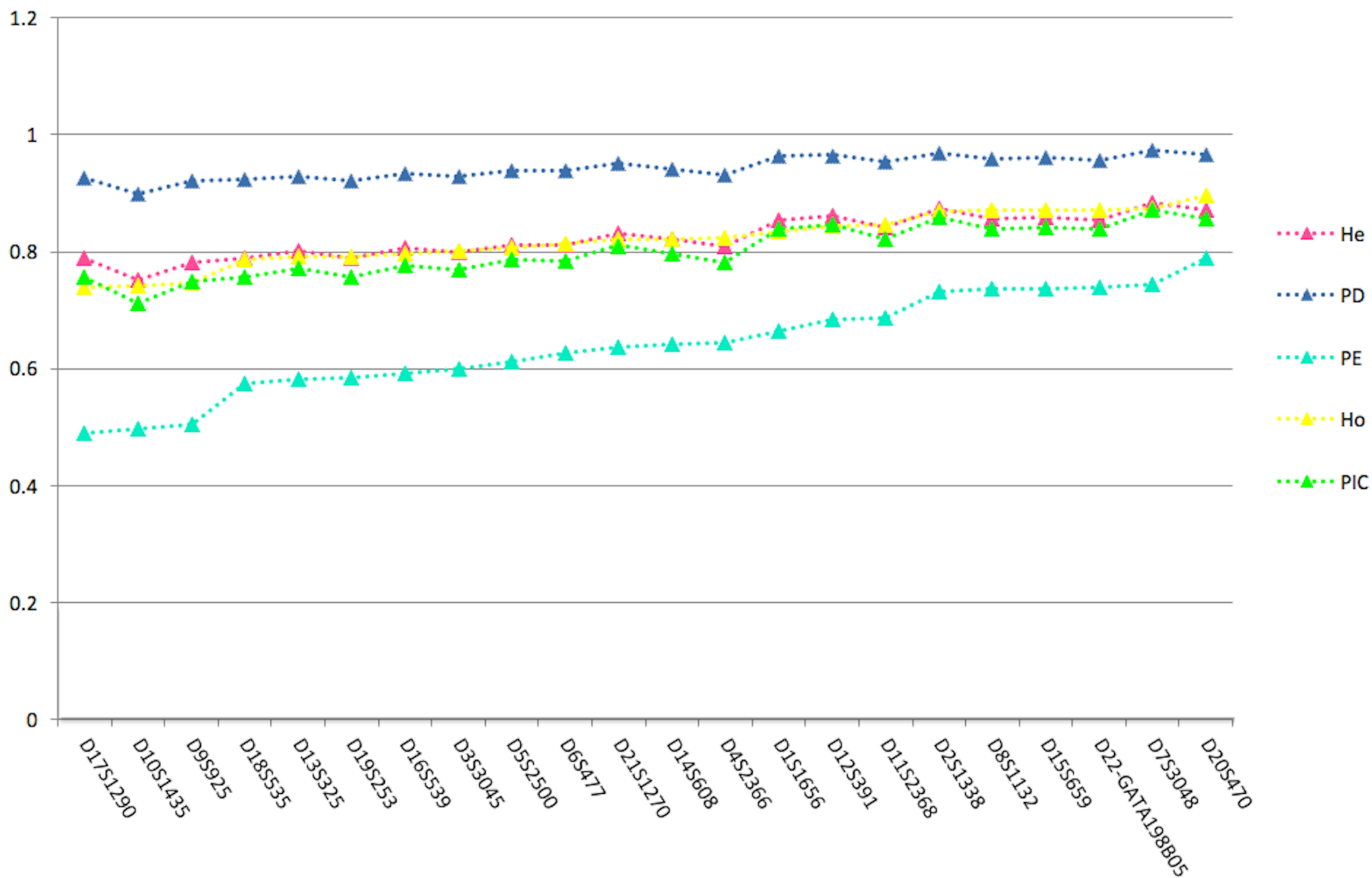

Supplement: Figure S3 [file peerj-07-6802-s007.pdf]
