# Supplementary material for: Forensic characteristics and population genetics of Chinese Kazakh ethnic minority with an efficient STR panel
Source: PeerJ. 2019 Apr 25;7:e6802. doi: 10.7717/peerj.6802 (PMC6487181; doi:10.7717/peerj.6802)
Supplement: Figure S1 [file peerj-07-6802-s005.pdf]

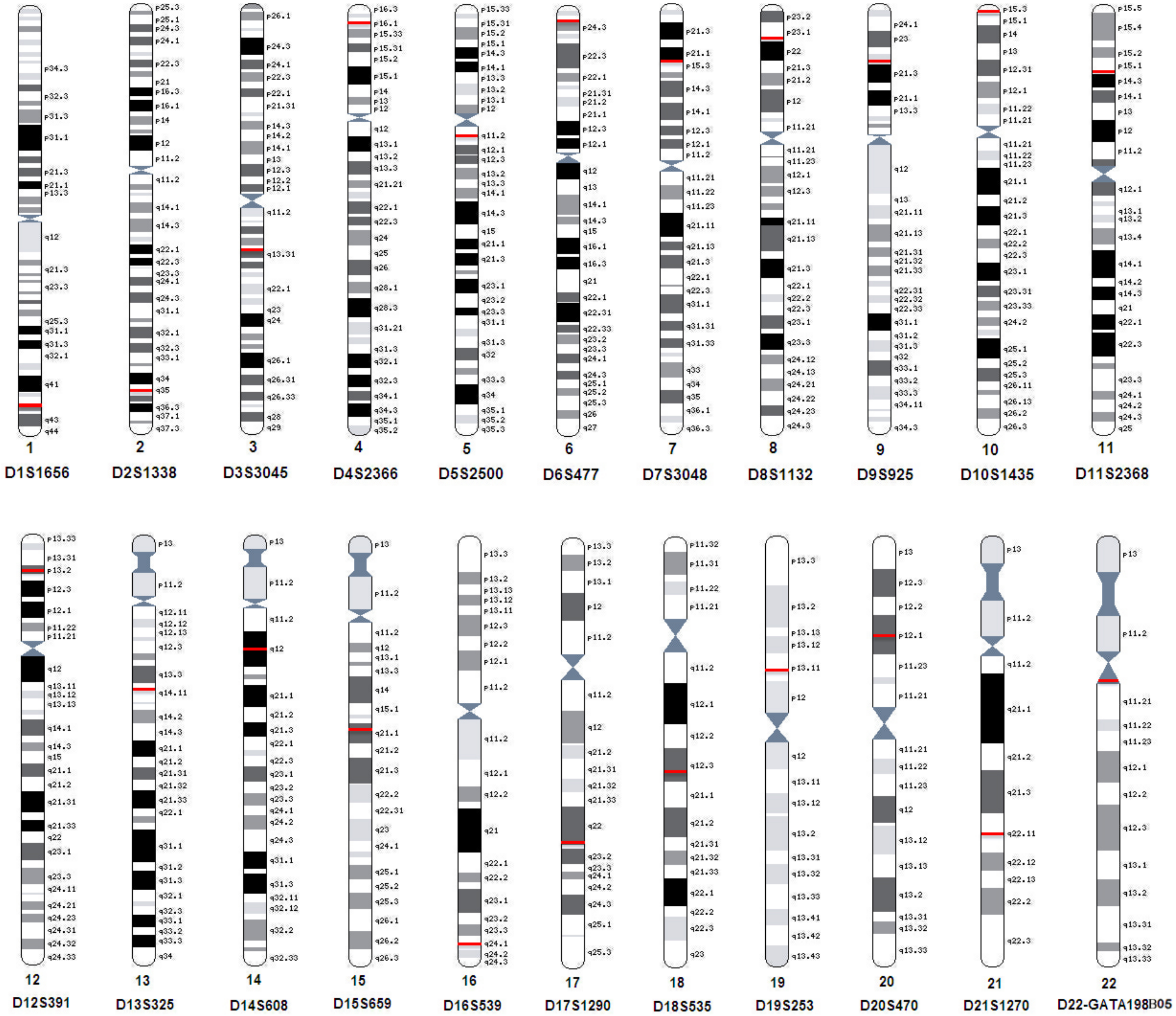
1
D1S1656
2
D2S1338
3
D3S3045
4
D4S2366
5
D5S2500
6
D6S477
7
D7S3048
8
D8S1132
9
D9S925
10
D10S1435
11
D11S2368
12
D12S391
13
D13S325
14
D14S608
15
D15S659
16
D16S539
17
D17S1290
18
D18S535
19
D19S253
20
D20S470
21
D21S1270
22
D22-GATA198B05
